# Influence of Owner Personality and Other Owner‐, Cat‐ and Treatment‐Related Factors on the Perception of Quality of Life in Cats With Hyperthyroidism

**DOI:** 10.1111/jvim.70091

**Published:** 2025-04-15

**Authors:** Sofie Muthmann, Imogen Schofield, Martin Kersting, Fabienne Blunschi, Joana Léonie Tiefenbrunner, Natali Betina Bauer, Katarina Hazuchova

**Affiliations:** ^1^ Clinic for Small Animals (Internal Medicine, Clinical Pathology and Clinical Pathophysiology) Justus Liebig University Giessen Germany; ^2^ CVS (UK) Ltd Norfolk UK; ^3^ Department of Psychology Justus Liebig University Giessen Germany

**Keywords:** big five Inventory‐2, hyperthyroidismCatQoL, QoL, radioiodine treatment

## Abstract

**Background:**

Assessment of quality‐of‐life (QoL) is becoming increasingly important in veterinary medicine. In human medicine, it is known that the assessor's personality might affect QoL.

**Objective:**

To evaluate the impact of owner personality and other owner, cat, and treatment‐related factors on the health‐related QoL (HRQoL) of hyperthyroid cats.

**Animals:**

Five hundred hyperthyroid cats.

**Methods:**

A prospective, cross‐sectional, questionnaire‐based study, conducted between April 2023 and February 2024. Owners completed the HyperthyroidismQoL‐cat and the Big Five Inventory‐2 to assess the cat's HRQoL and owner's five personality domains, respectively. Additional information about owner‐, cat‐, and treatment‐related factors was collected. Univariable and multivariable linear regression modeling was used to assess associations between owner personality, other factors, and the cat's HRQoL. Significance was *p* < 0.05.

**Results:**

In multivariable analysis, radioiodine treatment (RAIT; *p* = 0.001) and having a comorbidity (*p* ≤ 0.001) resulted in better HRQoL, whereas negative owner emotionality (*p* ≤ 0.001), having children ≤ 18 years of age (*p* = 0.04), treatment using a low iodine diet (LID; *p* = 0.023), no treatment (*p* = 0.03) and being hyperthyroid (*p* ≤ 0.001), hypothyroid (*p* = 0.004) or unknown thyroid status (*p* = 0.001) resulted in worse HRQoL.

**Conclusion and Clinical Importance:**

When interpreting HRQoL data, the potential impact of the personality domain negative emotionality (tendency to experience anxiety, fear, negative emotions) should be considered. Based on HRQoL, RAIT is the treatment of choice, whereas LID or no treatment are the least favored options. These findings should be considered when counseling owners about their cats' hyperthyroidism and its management.

**Abbreviations**
ATD: antithyroid drug treatment
BFI‐2: Big Five Inventory‐2
CKD: chronic kidney disease
FeLV: feline leukemia virus
FIV: feline immunodeficiency virus
HRQoL: health‐related quality‐of‐life
IQR: interquartile range
LID: low‐iodine diet
QoL: quality‐of‐life
RAI: radioactive iodine
RAIT: radioiodine treatment
TT4: total thyroxine

## 1 Introduction

Hyperthyroidism is the most common endocrine disease in middle‐aged to older cats, with a prevalence of approximately 10% in cats ≥ 10 years of age [1, 2, 3]. In most cases, the disease is caused by an overproduction of thyroid hormones due to thyroid adenoma, but a small proportion of cats might suffer from thyroid carcinoma [4]. Given the multisystemic effects of thyroid hormones, cats with hyperthyroidism exhibit a variety of clinical signs [5, 6], which, together with treatment and necessary monitoring, may negatively affect the quality of life (QoL) of both cats and their owners [6, 7].

Currently, four treatment options are available for hyperthyroidism in cats: radioiodine treatment (RAIT), thyroidectomy, antithyroid drugs (ATD), and low‐iodine diet (LID); of these, only the first two often are curative [8]. Treatment response traditionally has been assessed by measuring the total thyroxine (TT4) concentration, but assessment of QoL using a validated tool such as the HyperthyroidismCat–QoL [7] could represent a useful ancillary measure of evaluation of treatment success. Indeed, the use of QoL instruments to monitor the effectiveness of treatment or the negative influence of a disease is well established in people with hyperthyroidism [9, 10, 11] and many other conditions [12, 13, 14], and several disease‐specific [7, 15, 16, 17, 18, 19] or generic QoL [20, 21] tools also have been developed for dogs and cats.

When using QoL tools to assess the efficacy of a particular treatment in people, it is important to understand that a number of factors might impact the patient's assessment of his or her QoL. These factors might include the patient's personality [22, 23, 24] and socio‐demographic factors such as age, education, and employment status [22, 25, 26]. The effect of the assessor's personality has been demonstrated for both self‐ [22, 23, 27, 28] and proxy‐reported QoL [29, 30, 31]. In veterinary medicine, QoL assessment is conducted by a proxy person, the owner, or the veterinarian [32, 33]. However, the effect of owner personality or other owner‐related factors on QoL of the pet has, to our knowledge, not yet been investigated. Therefore, our aim was to evaluate whether and to what extent the assessment of health‐related quality of life (HRQoL) in cats with hyperthyroidism is influenced by the owner's personality. A second aim was to assess whether other owner‐, cat‐, and treatment‐related factors have an impact on the assessment of the HRQoL of hyperthyroid cats.

## 2 Materials and Methods

### 2.1 Study Design

Ours was a prospective, cross‐sectional, questionnaire‐based study conducted between April 2023 and February 2024 that assessed the effect of owner personality and other owner‐, cat‐, and treatment‐related factors on HRQoL of cats with hyperthyroidism. The study used the HyperthyroidismQoL‐cat [7] (Supporting Information: Appendix 1) to assess HRQoL of hyperthyroid cats, and the Big Five Inventory‐2 (BFI‐2 [34, 35]; Supporting Information: Appendix 2) to assess the personality of the cat owners. Additionally, socio‐demographic data about the owner and information about the cat and hyperthyroidism treatment were collected (Supporting Information: Appendix 3). All three questionnaires were made available online in both English and German, using an online survey program (LimeSurvey GmbH, Hamburg, Germany). Participants were informed about the purpose of the study and consented to participate by completing the questionnaire. Any hyperthyroid cat‐owner pair could participate, but incomplete questionnaires or those referring to multiple animals or deceased animals were excluded.

The survey was distributed through various channels, including primarily social media, cat‐related discussion forums, and website links.

### 2.2 HyperthyroidismQoL‐Cat

Health‐related QoL was evaluated using a questionnaire, the HyperthyroidismQoL‐cat [7], validated in both English and German (Supporting Information: Appendix 1). The questionnaire consists of 25 items grouped in four domains: a single owner‐related domain (6 items), and three cat‐related domains (19 items). The cat‐related domains are “gastrointestinal, dietary, urination” (7 items), “appearance” (3 items), and “activity and behavior” (9 items). Each item consists of 2 questions that determine the quantity (the frequency at which the item has an impact on owners' and cats' lives, on a scale 0–4) and quality (how strongly the item is perceived to affect HRQoL, on a scale 0–4) of the effect the item has on HRQoL. The score of each item is calculated as the product of the scores given for the two questions. The scores of all items then are added to form the total HRQoL score, ranging from 0 (the best possible HRQoL) to 382 (the worst possible HRQoL). When completing the HRQoL questionnaire, the owners were advised to relate their answers to the period of the past 4 weeks [7].

### 2.3 Big Five Inventory‐2

Assessment of the personality of the owner was performed using the BFI‐2 (Supporting Information: Appendix 2), a personality questionnaire validated in both English and German [34, 35]. The BFI‐2 is a commonly utilized instrument designed to measure five personality dimensions: extraversion, agreeableness, conscientiousness, negative emotionality, and open‐mindedness. These broad domains capture fundamental aspects of human personality. They have been shown to be reliable predictors of various behavioral and psychological outcomes.

According to previous work [36], openness to experience is defined by intellectual curiosity, imagination, and a willingness to challenge traditional beliefs. Individuals scoring high in this trait tend to be adventurous, innovative, and open to unconventional ideas. Conscientiousness reflects a tendency to be organized, disciplined, and goal‐oriented, often demonstrated through impulse control and socially responsible behavior. Extraversion is associated with being outgoing, energetic, and emotionally expressive, with extroverts gaining energy from social interactions. Agreeableness encompasses traits such as trust, altruism, and kindness, reflecting a cooperative and empathetic nature. Negative emotionality, previously termed neuroticism, refers to a predisposition toward negative emotions such as anxiety, anger, and emotional instability, with higher scores indicating higher sensitivity to stress [36].

Each of these five personality domains is formed by three subordinate facets (i.e., 15 facets in total). The BFI‐2 consists of 60 short questions (i.e., items), with five possible answers to each question, ranging from strongly disagree to strongly agree. An equal number of positive and negative questions (i.e., items with positive or negative loadings) are included. For positive items, the answers to each question are coded into the numbers 1 (= strongly disagree) to 5 (= strongly agree). For negative items, the answers are recoded (recoded value = 6 – [raw value]). Each of the 15 facets is formed from the average values of answers to four questions or items making up that respective facet. The domain, in turn, is calculated from the average of its three facets. In the end, the scores of the individual domains are obtained (each on a scale of 1–5) [34, 35].

### 2.4 Information About Owner, Cat, and Hyperthyroidism Treatment

In addition to the questions concerning the HRQoL and the owner's personality, general sociodemographic information about the owner such as age, sex, highest achieved education, presence of children < 18 years of age in the household, and current work situation was assessed. Furthermore, the owners were asked about the number of cats in the household, whether the owner completing the survey is the primary care‐giver, how many cats are in the household and whether this cat is the owner's first cat. Information on the cat's signalment, severity of clinical signs of hyperthyroidism, treatment for hyperthyroidism, and presence of comorbidities (e.g., chronic kidney disease [CKD] and heart disease) and their treatment was obtained. The owners were asked about the cat's thyroid status (eu‐/hypo‐/hyperthyroidism/unknown), but information on the specific TT4 concentration was not collected. The wording of these additional questions can be found in Supporting Information: Appendix 3.

### 2.5 Statistical Analysis

Data were downloaded from LimeSurvey as Excel files (Excel, Microsoft Corp, USA). Excel also was used to calculate the HRQoL and personality scores. Stata 17.0 (Stata, TX, USA) was used for statistical analysis. Data were assessed for normality using the Shapiro–Wilk test and visual analysis of histograms. Normally distributed data are presented as mean ± SD, non‐normally distributed data as median (interquartile range [IQR]). Categorical data are presented as numbers and percentages (proportion).

Univariable and multivariable linear regression modeling was used to assess associations between owner personality scores and other sociodemographic, owner‐ and cat‐related factors, and the cat's HRQoL score. Risk factors with a broad association with HRQoL during univariable analysis (likelihood ratio test = *p* < 0.2) were deemed potential confounding factors and were taken forward to multivariable evaluation. Multivariable model building used a backward stepwise manual approach [37]. Potential pairwise correlations were explored to identify potential colinearity using Spearman's rho and were considered highly correlated if rho > 0.80. If two variables were considered biologically plausible collinear variables, such as the presence of comorbidities and the presence of CKD, the variable with the largest sample size was taken forward to multivariable analysis. Univariable restricted cubic spline models were used to assess the assumption of linearity with the logit. Furthermore, a post hoc analysis using univariable linear regression was performed to analyze the influence of the facets of the personality domain negative emotionality on HRQoL score and to explore the relationship between the personality domain negative emotionality and the different HRQoL tool domains. Non‐normally distributed continuous variables were transformed into normal distribution using square root transformation for both uni‐ and multivariable modeling. Transformation was not needed for post hoc analysis. Statistical significance was set at *p* < 0.05.

## 3 Results

### 3.1 Information About Cat Owners

In total, 500 valid questionnaire responses were obtained, of which 283/500 (57.6%) were completed in German and 217/500 (43.4%) in English. The highest proportion of owners resided in Germany (*n* = 131/500, 26.2%), followed by the United States (*n* = 116/500, 23.2%), whereas 151/500 (30.2%) did not disclose their country of residence. Two hundred twenty‐two (*n* = 222/500, 44.4%) owners were > 50 years old, 113/500 (22.6%) were between 41 and 50 years old, 117/500 (23.4%) were between 31 and 40 years old, 39/500 (7.8%) were < 30 years old, and 9/500 (1.8%) did not disclose their age. Almost all participants (*n* = 470/500, 94%) were female, whereas 26/500 (5.2%) were male, and 4/500 (0.8%) did not disclose their sex. Approximately one‐third (*n* = 178/500, 35.6%) worked full‐time; the most common highest educational qualification was a bachelor's degree (*n* = 116/500, 23.2%). Only 64/500 (12.8%) owners had children < 18 years of age. The remaining information about the sociodemographic background of the owners can be found in Supporting Information: Appendix 4.

Most owners completing the questionnaire (*n* = 448/500, 89.6%) were the primary care‐giver of the cat about which the questionnaire was answered, and 250/500 (50%) spent > 7 h per day with their cat. For 398/500 (79.6%) owners, this cat was not their first cat, and 82/500 (16.4%) already had managed a cat with hyperthyroidism in the past. At the time of questionnaire completion, approximately one‐third of the participants each had one (*n* = 157/500, 31.4%), two (*n* = 157/500, 31.4%) or three or more cats (*n* = 182/500, 36.4%; no answer: *n* = 4/500, 0.8%).

### 3.2 Information About the Cats

Half of the cats (*n* = 250/500, 50%) were between 11 and 14 years of age, whereas 197/500 (39.4%) were > 15 years old. The sex was evenly distributed, and domestic/European shorthair cats were most frequently represented (*n* = 338/500, 67.6%). Additional details about the signalment are presented in Table 1.

**TABLE 1:** Age, sex, and breed of the 500 hyperthyroid cats included in the study.

Parameter/question	Choice of options	Number *n* (%)
Age	3–6 years	5 (1)
	7–10 years	43 (8.6)
	11–14 years	250 (50)
	Over 15 years	197 (39.4)
	Unknown	5 (1)
Sex	Male	10 (2)
	Male neutered	245 (49)
	Female	17 (3.4)
	Female neutered	228 (45.6)
Breed	Domestic/European shorthair	338 (67.6)
	Mixed breed	56 (11.2)
	Maine Coon	10 (2)
	Norwegian forest	10 (2)
	British shorthair	9 (1.8)
	Domestic longhair	9 (1.8)
	Siamese	6 (1.2)
	Persian	3 (0.6)
	Ragdoll	3 (0.6)
	Bengal	1 (0.2)
	Others	36 (7.2)
	Unknown	19 (3.8)

Indoor cats made up 57.8% (*n* = 289/500) of the study cohort. The cats most commonly had been hyperthyroid for 1–2 years (*n* = 174/500, 34.8%), and almost a quarter (*n* = 112/500, 22.4%) for ≥ 3 years. On the other hand, 132/500 (26.4%) cats had been hyperthyroid for < 6 months and 53/500 (10.6%) for 6 to 12 months. The duration of the disease was unknown in 29/500 (5.8%) cases. Table 2 provides an overview of the treatment options for hyperthyroidism known to the owners and those that were used in the included cats. Most cats were treated using ATD, which was also the treatment option about which almost all owners were aware.

**TABLE 2:** Treatment options for hyperthyroidism that the owners were aware of as well as the treatment options used in the 500 cats with hyperthyroidism.

Treatment option	Treatment options the owners were aware of	Current treatment
	*n* (%)	*n* (%)
Antithyroid tablets/sirup	488 (97.6)	343 (69)
Antithyroid ear ointment	388 (77.6)	63 (12.6)
Radioiodine treatment	427 (85.4)	48 (9.6)
Low iodine diet	378 (75.6)	6 (1.2)
Surgery/thyroidectomy	353 (85.4)	5 (1)
No treatment	20 (4)	22 (4.4)
Other	—	13 (2.6)

**FIGURE 1:**
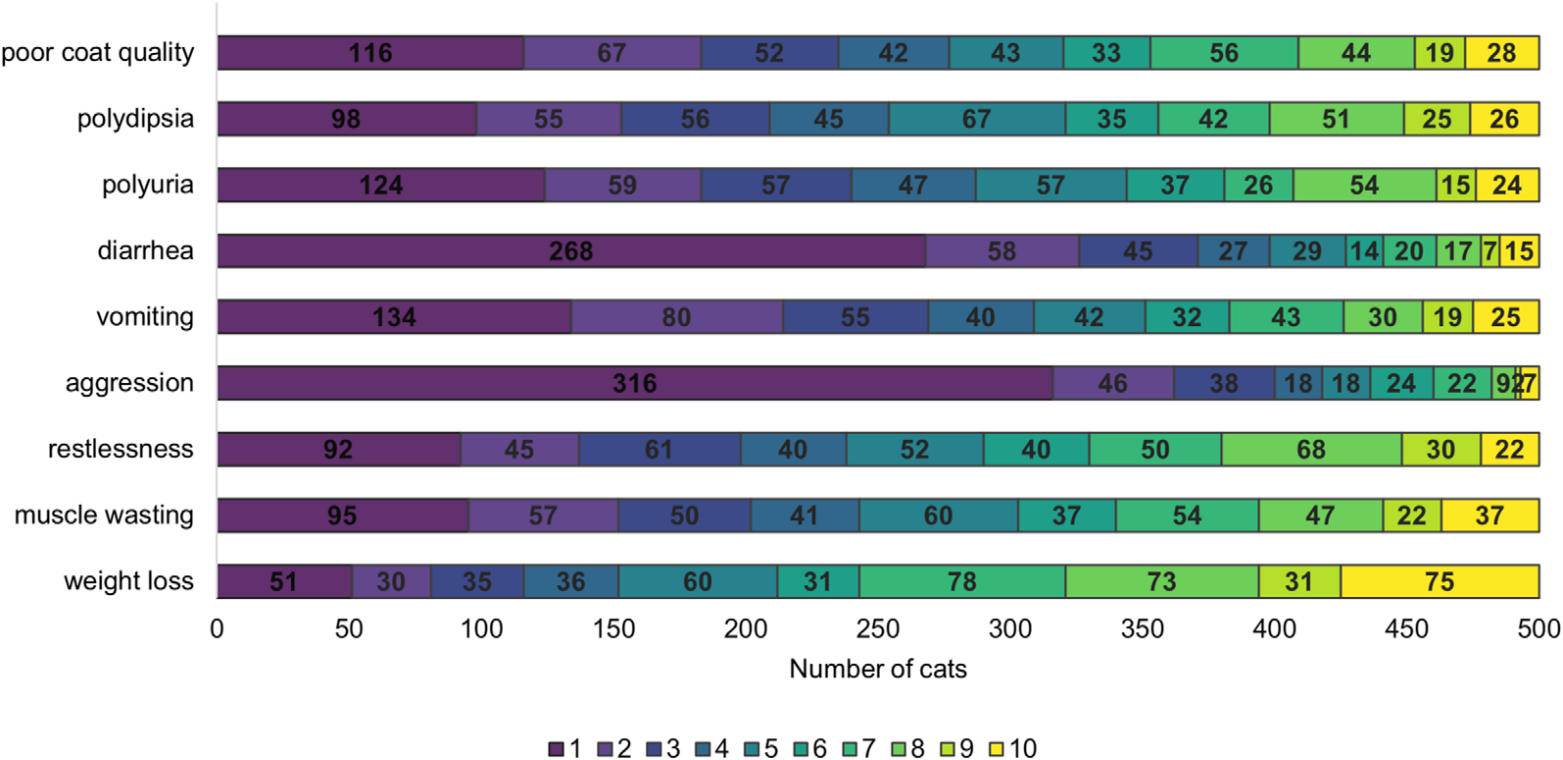
Clinical signs of hyperthyroidism as stated by the owners of 500 cats at the time of the survey completion (rated on a scale from 1 to 10 [1 = not present to 10 = very strong]). The different numbers on the scale from 1 to 10 are color‐coded (legend for the color scheme is within the figure) and the number of cats for which the owner stated the respective severity for each of the clinical signs is within the respective bar.

The primary reason of most owners for their current treatment choice was their primary care veterinarian's advice (*n* = 323/500, 64.6%); other reasons were internet research (*n* = 35/500, 7%), personal experience (*n* = 34/500, 6.8%), advice from friends and acquaintances (*n* = 26/500, 5.2%), and drug intolerances (*n* = 18/500, 3.8%). Of all cats treated for their hyperthyroidism (*n* = 478/500, 95.6%), 169/478 (35.4%) had been treated for < 6 months, 52/478 (10.9%) had been treated for 6 months to 1 year, 52/478 (10.9%) for 1–2 years, and 190/478 (39.7%) for ≥ 2 years. No information about treatment duration was provided by 25/478 (5.2%) owners. The owners were asked to state how marked or severe the clinical signs of hyperthyroidism were in their cats at the time of questionnaire completion (Figure 1). Most clinical signs besides weight loss were not marked in the study cohort.

**FIGURE 2:**
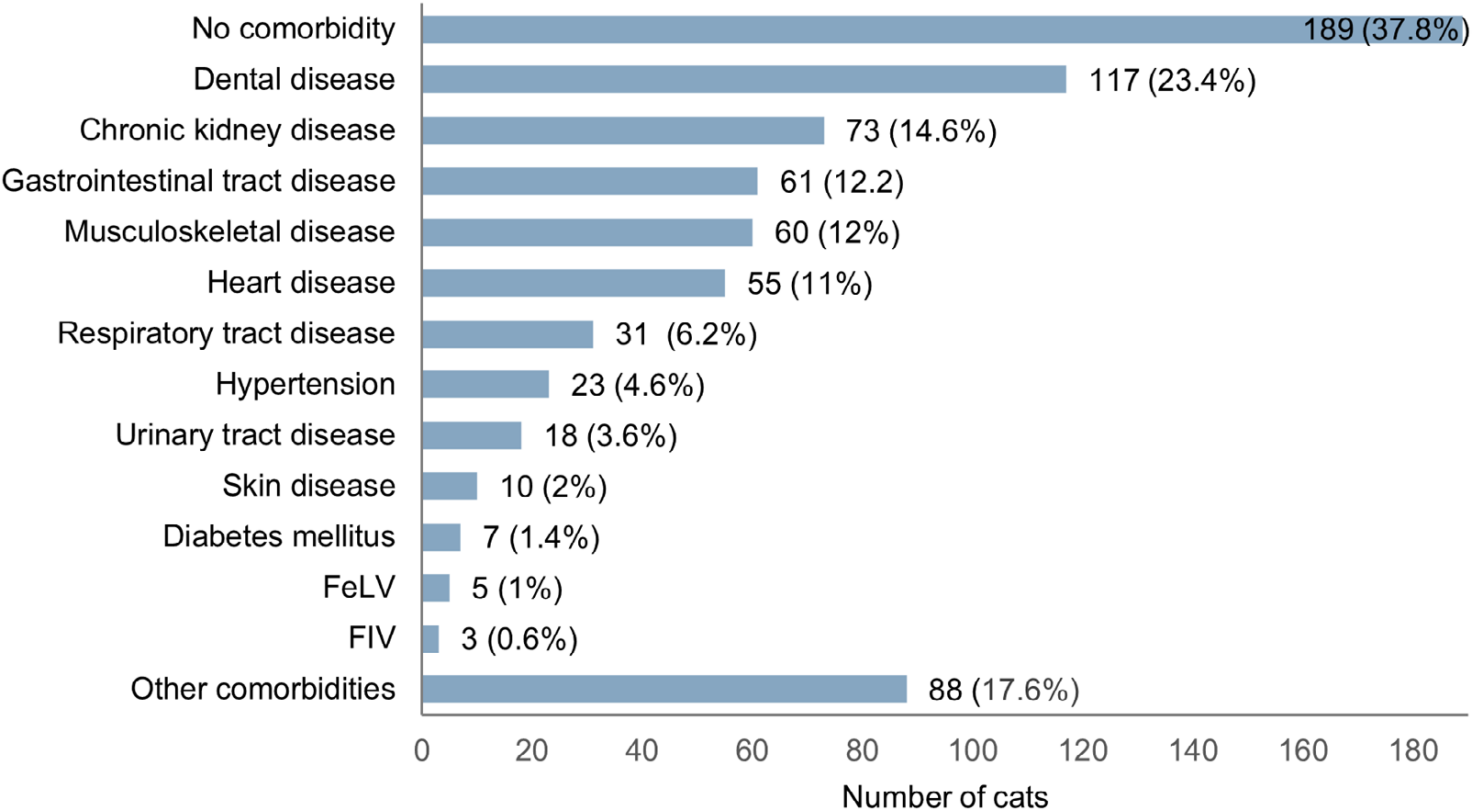
Number (%) of cats with and without comorbidities among the 500 hyperthyroid cats included in the study. Each bar represents number (% out of 500) of cats with a specific comorbidity (or without any comorbidity). FeLV: feline leukemia virus, FIV: feline immunodeficiency virus.

**FIGURE 3:**
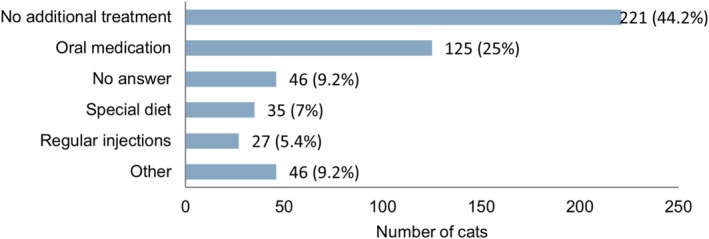
Number (% out of 500 cats participating in the study) of cats that were receiving some form of treatment in addition to their hyperthyroidism treatment.

Concerning thyroid status at the time of the survey, 273/500 (54.6%) of cats were euthyroid, 124/500 (24.8%) were hyperthyroid, 25/500 (5%) were hypothyroid, and 78/500 (15.6%) of the owners were unaware of their cats' current thyroid status. Approximately a quarter of the cats (*n* = 129/500, 25.8%) had their thyroid results checked four times, 85/500 (17%) three times, 116/500 (23.2%) two times, and 54/500 (10.8%) once a year. Over one‐third of the cats (*n* = 89/500, 37.8%) had no other known disease besides hyperthyroidism, whereas one‐third (*n* = 166/500, 33.2%) had one comorbidity, and 112/500 (24.4%) had ≥ 2 comorbidities. An overview of the prevalence of the various comorbidities is presented in Figure 2. Treatments that cats were receiving for their comorbidities are presented in Figure 3.

### 3.3 HRQoL Scores of the Cats and Owner Personality Scores

The median HRQoL score was 84 (IQR, 56–116; range, 3–278) and the owners' perceived overall QoL of their cats was very good for 35.6% (*n* = 178/500), good for 41.2% (*n* = 206/500), satisfying for 16.2% (*n* = 81/500), poor for 5.8% (*n* = 29/500), and very poor for 1.2% (*n* = 6/500). For linear regression, HRQoL was transformed using a square root transformation [38] to meet the assumptions of the model.

Mean personality scores obtained from the BFI‐2 were: extraversion: 3.11 (SD, 0.70), agreeableness: 3.87 (SD, 0.53), conscientiousness: 3.55 (SD, 0.60), negative emotionality: 2.91 (SD, 0.67), and open‐mindedness: 3.61 (SD, 0.73). The scores for the 15 subordinate facets of the BFI‐2 and their comparison with the reference values of the German‐ and English‐speaking population [34, 35] can be found in Supporting Information: Appendix 5. There was a negligible to weak negative correlation between negative emotionality and all other personality domains (open mindedness: rho = −0.09, *p* = 0.04; agreeableness: rho = −0.18, *p* ≤ 0.01; conscientiousness: rho = −0.31, *p* ≤ 0.01; extraversion: rho = −0.38, *p* ≤ 0.01). No significant correlation was found between the other domains (all *p* > 0.05). Negative emotionality and owner age were strongly associated (Chi‐square test: *p* = 0.007).

### 3.4 Univariable Analysis

The results of the univariable analysis are presented in Table 3.

**TABLE 3:** Results of the univariable analysis assessing the effect of owner personality and possible confounding factors on the HRQoL.

Variable	Category	Regression‐coefficient	95% Confidence interval	*p*
Owner personality				
Extraversion	—	−0.19	−0.53 to 0.15	0.27
Agreeableness	—	−0.16	−0.61 to 0.28	0.47
Conscientiousness	—	−0.25	−0.64 to 0.15	0.22
Negative emotionality	—	0.89	0.55–1.24	**< 0.001**
Open‐mindedness	—	−0.11	−0.43 to 0.21	0.5
Possible confounding factors				
Owner gender	Female*			
	Male	0.62	−0.44 to 1.69	0.25
	Prefer not to say	0.97	−1.69 to 3.62	0.48
Owner age	30 years or younger*			
	31–40 years	−0.12	−1.09 to 0.85	0.81
	41–50 years	−0.76	−1.74 to 0.21	**0.12**
	50 years or older	−1.09	−2.01 to −0.18	**0.02**
Having children 18 or younger living in the same household	No*			
	Yes	0.61	0.03–1.20	**0.04**
Estimated time spent with cat per day	Less than 7 h per day*			
	More than 7 h per day	−0.34	−0.82 to 0.15	**0.18**
Is this hyperthyroid cat the first cat of the owner	No*			
	Yes	0.35	−0.24 to 0.93	0.25
Number of cats owned at the moment	One cat*			
	Two cats	−0.44	−1.03 to 0.16	**0.15**
	Three or more cats	−0.80	−1.37 to −0.23	**0.01**
Had the owner had a cat with hyperthyroidism previously	No*			
	Yes	−0.58	−1.22 to 0.05	**0.07**
Cat's age	7–10 years*			
	3–6 years	−1.32	−3.82—1.17	0.3
	11–14 years	−0.23	−1.10 to 0.64	0.6
	15 years or older	0.25	−0.64 to 1.14	0.58
	Unknown	0.31	−2.19 to 2.80	0.81
Duration of hyperthyroidism	< 3 months*			
	4–6 months	0.07	−0.86 to 0.99	0.89
	7 months—1 year	−0.39	−1.38—0.60	0.44
	1–2 years	−0.44	−1.22 to 0.34	0.27
	3–4 years	−0.02	−0.93 to 0.88	0.96
	5 years or longer	−0.02	−0.93 to 1.10	0.98
	Unknown	−0.24	−1.43 to 0.96	0.7
Cat's hyperthyroidism treatment	Antithyroid tablets/syrup*			
	Ear ointment	0.11	−0.59 to 0.81	0.76
	Low iodine diet	2.66	0.55–4.77	**0.01**
	Radioiodine treatment	−1.62	−2.41 to −0.83	**< 0.001**
	Surgery	0.15	−2.16 – 2.45	0.9
	No treatment	1.94	0.81–3.01	**0.001**
	Other	1.03	−0.41 to 2.48	**0.16**
Thyroid status	Euthyroid*			
	Hypothyroid	1.02	−0.03 to 2.06	**0.06**
	Hyperthyroid	2.10	1.55–2.64	**< 0.001**
	Unknown	1.07	0.43–1.71	**0.001**
Comorbidities	No*			
	Yes	−1.10	−1.58 to −0.62	**< 0.001**
Chronic kidney disease (CKD)	No CKD*			
	CKD	1.27	0.61–1.94	**< 0.001**

*Note:* For each confounding factor, the reference category is listed first and is marked with an asterisk. Factors with a broad association *p* < 0.2 are shown in bold letters.

Of the five personality domains, only negative emotionality had a significant influence. The following variables had a broad association with the HRQoL score: negative emotionality, owner age (41–50 years and ≥ 50 years old), having children ≤ 18 years old in the household, spending > 7 h per day with the cat, number of cats owned at the moment (2 cats and ≥ 3cats), if the owner had a cat with hyperthyroidism before, hyperthyroidism treatment modality (LID, RAIT, no treatment, and other), thyroid status, having ≥ 1 comorbidity, and having CKD.

### 3.5 Multivariable Analysis

The following variables were taken forward to multivariable modeling: negative emotionality, owner age, having children ≤ 18 years old in the household, hyperthyroidism treatment modality, comorbidities, and thyroid status. The number of cats in the household was removed from the model during the backward stepwise approach to model building, as was time spent with the cat and if the owner had a cat with hyperthyroidism previously because these three variables no longer remained significant. The results of the multivariable analysis are shown in Table 4.

**TABLE 4:** Results of the multivariable analysis of associations with the square root of the health‐related quality‐of‐life score in cats with hyperthyroidism.

Variable	Category	Regression‐coefficient	95% Confidence interval	*p*
Owner negative emotionality	—	0.75	0.42–1.08	**< 0.001**
Owner age	30 years or younger*			
	31–40 years old	0.35	−0.53 to 1.23	0.44
	41–50 years old	−0.26	−1.15—0.63	0.56
	50 years or older	−0.32	−1.16 to 0.51	0.45
Children 18 or younger	No*			
	Yes	0.56	0.02–1.10	**0.04**
Cat's hyperthyroidism treatment	Antithyroid tablets/syrup*			
	Ear ointment	−0.03	−0.68 to 0.63	0.94
	Diet	1.23	−0.75 to 3.21	**0.02**
	Radioiodine treatment	−1.47	−2.22 to −0.72	**< 0.001**
	Surgery	−0.48	−2.62 to 1.67	0.66
	No treatment	1.19	0.12–2.26	**0.03**
	Other	1.05	−0.55 to 2.15	0.24
Thyroid status	Euthyroid*			
	Hypothyroid	1.50	0.49–2.51	**0.004**
	Hyperthyroid	1.70	1.16–2.24	**< 0.001**
	Unknown	1.09	0.57–1.71	**0.001**
Comorbidity	No*			
	Yes	−0.85	−1.29 to −0.40	**< 0.001**

*Note:* For each confounding factor, the reference category is listed first and is marked with asterisk. Significant *p* values are shown in bold letters.

Multivariable analysis identified a significant association with the square root of the HRQoL score for negative emotionality; having children ≤ 18 years old; treatment with RAIT, LID, and no treatment; hyper‐ and hypothyroidism as well as unknown thyroid status; and, the presence of comorbidities. Radioiodine treatment and having a comorbidity resulted in a lower (better) HRQoL score, whereas negative emotionality, having children ≤ 18 years old, treatment with LID or no treatment as well as hyper‐ or hypothyroidism and unknown thyroid status were associated with a higher (worse) HRQoL score.

### 3.6 Post Hoc Analysis

Post hoc analysis was performed to assess which facet of the domain “negative emotionality” was most influential on the association between negative emotionality and HRQoL. It indicated that all three facets, which form the dimension negative emotionality, had a significant influence on the HRQoL (Table 5).

**TABLE 5:** Results of the post hoc analysis showing the influence of the three facets of the domain negative emotionality on the HRQoL score.

Facet	Regression coefficient	95% Confidence interval	*p*
Negative emotionality			
Anxiety	0.59	0.32–0.85	**< 0.001**
Depression	0.63	0.39–0.87	**< 0.001**
Emotional volatility	0.52	0.10–0.94	**0.01**

*Note:* Significant *p* values are shown in bold letters.

Post hoc analysis also was performed to assess if any of the HRQoL domains was more strongly associated with the personality domain “negative emotionality.” This analysis indicated that the HRQoL domain “owner” had the strongest association with negative emotionality, whereas no association was found with the domain “appearance” (Table 6).

**TABLE 6:** Post hoc univariable analysis of the relationship of negative emotionality with different HRQoL tool domains, with significant *p* values shown in bold letters.

HRQoL domain	Regression coefficient	95% Confidence interval	*p*
Owner	8.79	6.43–11.15	**< 0.001**
Dietary, gastrointestinal, urination	3.62	1.50–5.75	**0.001**
Appearance	0.61	−0.52 to 1.75	0.29
Behavior	4.00	0.96–7.00	**0.01**

## 4 Discussion

Our study identified that the negative emotionality of the owner (describing the tendency to experience anxiety, fear, and other negative emotions) had a significant effect on HRQoL of hyperthyroid cats assessed using the Hyperthyroidism QoL‐cat tool [7], with increasing negative emotionality scores being associated with higher HRQoL scores and therefore worse HRQoL. Other factors associated with worse HRQoL in this study were having children ≤ 18 years old, treatment with LID or no treatment, and hyper‐ or hypothyroidism or unknown thyroid status under treatment of hyperthyroidism. On the other hand, RAIT and the presence of comorbidities were associated with better HRQoL (lower HRQoL scores).

Our study determined that higher scores in the owner personality domain “negative emotionality” were associated with worse HRQoL in cats with hyperthyroidism. The association between personality traits (including “negative emotionality”) and HRQoL has been demonstrated in several chronic conditions in people, including mood and anxiety disorders [24], inflammatory bowel disease [27], breast cancer [23], and CKD [39], and also was identified in a recent systematic review [40]. This systematic review included 76 studies that examined the relationship of personality characteristics with HRQoL and found that some personality characteristics such as “negative emotionality” were consistently associated with psychosocial aspects of the HRQoL, whereas their effect on physical aspects of HRQoL was less pronounced [40]. Similarly, in our study, the strongest association with “negative emotionality” was identified for the HRQoL domain “owner,” whereas the association with the other domains was weaker or not significant. Indeed, the “owner” domain focuses on the owner's concerns regarding his or her cat's hyperthyroidism and its treatment (i.e., psychosocial aspects of HRQoL), whereas the other three domains relate more to the physical aspects (i.e., clinical presentation) of hyperthyroidism. On the other hand, although the association between “negative emotionality” and HRQoL‐domains “gastrointestinal, dietary, urination” and “behavior” was somewhat weaker, it still was significant. This finding is in agreement with the results of a previous study that investigated the relationship between the personality of the owner and the lifestyles to which cats are exposed (e.g., indoor/outdoor), cat behavior and well‐being [41]. That study found that higher owner scores for “negative emotionality” (termed “neuroticism” in that study) were significantly associated with cats having a preexisting medical condition, displaying more frequent stress‐related sickness behavior, behavioral problems and aggressive or anxious behavior [41]. Although causal mechanisms for the findings of that study could not be determined, the authors discussed that owners with high “negative emotionality” scores could either negatively affect their cats' behavior, be more likely to select cats with behavioral characteristics similar to their own, or merely be more worried or pessimistic about their cats' behavior. They further postulated that because personality traits are considered stable, it is unlikely that it is the cat's behavior that negatively affects its owner [41]. Although we cannot prove this hypothesis, our interpretation of the effect of the negative emotionality of the owners on HRQoL of their hyperthyroid cats in our study also would be in favor of owners with high “negative emotionality” scores rating their cats' HRQoL worse than it truly is. This effect also has been shown in humans (e.g., in elderly persons with higher levels of “negative emotionality” who self‐reported inferior hearing and motor functioning than indicated by their results at several performance‐based tests [42]). Considering the results of these studies indicating the negative effect of “negative emotionality” on HRQoL assessment, it was suggested that understanding patient personality characteristics might help in developing strategies to improve treatment adherence and health outcomes in people [40]. In veterinary medicine, knowledge of the owner's personality also could be potentially helpful in improving owner counseling regarding their cat's condition (e.g., hyperthyroidism) and its treatment. However, when interpreting the influence of owner personality on a cat's HRQoL, it is important to understand the magnitude of the effect of “negative emotionality” on the HRQoL score. Although the scores for personality domains range from 1 to 5, the cat HRQoL score might range from 0 (best HRQoL) to 382 (worst HRQoL); in our study the median HRQoL was 84. When interpreting the relationship between these two scores, we must bear in mind that our study identified an association between “negative emotionality” scores and the square root of HRQoL scores rather than the “raw” HRQoL scores (the “raw” HRQoL scores had to be normalized to meet the assumptions of the model). The following example might help illustrate the magnitude of the effect: if the difference in ‘negative emotionality’ scores between two owners were 1 (i.e., one owner having a score of 2.5 and the other of 3.5), this would increase the square root of the HRQoL score given by the owner with higher negative emotionality 0.75 times, meaning that, for example, an HRQoL score of 136 would increase to 154. Although not a large change on a scale of 0–382, a difference in the personality score of 1 is relatively large [34]. Because less marked differences in personality scores are more likely than such large deviations [34], the effect of negative emotionality of the owner on the total HRQoL of hyperthyroid cats in real life is likely subtle [43].

Interestingly, owners with children < 18 years old perceived their cats' HRQoL worse than owners without children. One possible explanation for this phenomenon might be found in the dual caregiving demands of children and pets increasing the emotional and financial burden for the owner. A sick pet in a household with children can disrupt established family routines and might contribute to increased stress levels, particularly related to frequent veterinary visits and daily administration of medication. These challenges might leave owners with fewer resources to adapt effectively to their cats' needs [43, 44, 45].

Besides the effect of owner personality and owner‐related factors on the QoL assessment, the effect of cat‐ and treatment‐related factors also was assessed. When comparing the different treatment options, it is not surprising that the cats without treatment had a significantly worse QoL compared with cats receiving ATD (tablets or syrup). Cats not (yet) treated for their hyperthyroidism probably had more marked clinical signs of the disease, which likely was reflected in their owners' assessment of HRQoL items included in the three cat‐centered domains. Insufficient control of clinical signs also could be the reason for the finding of worse HRQoL in cats fed LID when compared with cats receiving ATD. Previous research has shown that cats fed LID had no reduction in heart rate and no significant weight gain, despite the fact that most of these cats achieved euthyroidism [46]. Of note, all six LID‐fed cats were hyperthyroid at the time of study participation, despite being fed the LID for a minimum of 2 months (which is the time needed for the diet to achieve its full effects). Although the statistical model accounted for thyroid status and therefore feeding LID was associated with HRQoL regardless of being hyperthyroid, a larger cohort of cats should be analyzed, ideally also including euthyroid LID‐fed cats, to allow a more robust assessment of feeding LID on HRQoL of hyperthyroid cats.

In contrast to previous studies using the HyperthyroidismQoL‐cat tool [7, 47], in our study, radioactive iodine (RAI) treated cats had significantly better QoL than cats treated with ATD (tablets or syrup). Treatment with RAI would be expected to decrease the square root of the HRQoL score 1.47 times in comparison to ATD, meaning that, for example, a cat with an HRQoL score of 80 receiving ATD would be expected to have an HRQoL score of 56 when treated with RAI. Although this is what one would expect with curative treatment, a previous study that compared changes in HRQoL between cats treated with ATD (enrolled into the study within 6 months of treatment start) and RAI‐treated cats over a 6‐month period after RAIT found no difference in QoL between these groups [47]. However, the lack of difference between the treatment groups in that study might have been a consequence of the small number of included cats (15 cats treated with ATD and 23 with RAIT) [47]. Furthermore, the follow‐up in that previous study was relatively short (6 months post‐RAIT) [47], whereas some of our cats had been treated with RAIT several years ago. Although in most RAI‐treated cats hyperthyroidism resolves within 2 weeks of treatment, in individual cats resolution might take > 6 months [48]. In addition, studies in humans have shown that HRQoL might be decreased for years even after normalization of thyroid hormone concentrations [11, 49]. Therefore, the different results of our study and the previous study [47] might be associated with the longer time period between RAIT and the HRQoL assessment in our study. A study that designed and validated the HyperthyroidismQoL‐cat tool used in our study also found no effect of treatment modality (32/225 hyperthyroid cats in that study were treated with RAI) on HRQoL [7]. However, in approximately 50% (*n* = 17/32) of the RAI‐treated cats evaluated in that study, the treatment was conducted < 6 months previously, and thyroid status was not assessed and therefore not accounted for in the statistical analysis [7]. On the other hand, a positive effect of RAIT on QoL of hyperthyroid cats, similar to our findings, also was identified in a previous survey [50]. That study, however, did not use a validated QoL tool [50]. Although our study used a more robust multivariable statistical model than previous studies that used the HyperthyroidismQoL‐cat tool [7, 47], given their contradictory results, additional studies evaluating the long‐term QoL of cats treated with RAIT might shed light on the effect of RAIT (and other treatment modalities) on the HRQoL of hyperthyroid cats.

Not surprisingly, hyperthyroid state was associated with worse HRQoL in our study. This finding is likely related to the lack of control of clinical signs, as discussed above. However, hypothyroid state was also associated with worse QoL. Although iatrogenic hypothyroidism secondary to hyperthyroidism treatment might be associated with only mild or no clinical signs [51], this condition might lead to the development of azotemia, and azotemic hypothyroid cats have shorter survival times when compared with those without azotemia [52]. Therefore, deterioration of renal function could be the reason for the worse HRQoL in the hypothyroid cats, although this hypothesis cannot be confirmed based on our study because renal function was not assessed. In some cats, treatment of hypothyroidism or development of clinical signs of hypothyroidism also might have affected their HRQoL. Neither the treatment nor the clinical signs of hypothyroidism were assessed in our study. In addition, HRQoL also was worse in cats with unknown thyroid status. The reason for this finding is difficult to understand, but possibly the clinical signs in these cats might not have been well controlled, or the owners might have been distressed and their lives negatively affected by their cats' disease and its treatment.

Surprisingly, cats suffering from another condition in addition to hyperthyroidism had a better HRQoL than those without comorbidity. This finding contradicts the results of a previous study that found worse HRQoL in cats with comorbidities than in cats suffering from hyperthyroidism alone [7]. The reason for the discrepant results of these two studies is unknown, but intuitively one might expect worse QoL in cats with multiple diseases. In both studies, several different comorbidities were identified, but neither the severity nor the quality of the control of these comorbidities was assessed and taken into account. Furthermore, the question concerning comorbidities was quite open, and the definition of a comorbidity was not provided to the owners. Therefore, our (and the previous) [7] study relied on the owners' knowledge of their cats' concurrent conditions, without having access to medical records. A possible reason for the positive effect of comorbidities on HRQoL in our study could be that cats with comorbidities might be presented more frequently for veterinary re‐evaluations, which allows for early identification of comorbidities as well as the institution of appropriate treatment. If cats without comorbidities actually were presented to their veterinarians less frequently, any concurrent conditions might have gone unnoticed and therefore not treated, possibly negatively affecting HRQoL. The impact of veterinary re‐evaluations on HRQoL, however, could not be assessed in our study because the frequency of veterinary visits other than those for assessment of thyroid function was not evaluated.

Our study had some limitations. It is possible that sampling biases [53] inherent to the study might have influenced the results. Because all participants were aware of the study aim, some might have been tempted to choose answers that were more in line with how they wanted their personality and the HRQoL of their cat to be [54]. Additionally, the survey was only available online, and most participants were made aware of the study through social media, specialized Facebook groups for feline hyperthyroidism, or cat forums. People with no regular access to the internet likely are underrepresented. Furthermore, most participants probably already had a heightened interest or concern about their cat's hyperthyroidism and therefore were more likely to respond to our survey. Therefore, the study cohort might not represent the average cat owner. Because 470/500 (94%) of the participants were female, our study cannot make a general statement about male owners. In addition, the treatment options for hyperthyroidism were not evenly distributed because most cats were treated with ATD. Although this result likely reflects the treatment modalities used in veterinary practice, it makes it difficult to make general statements about underrepresented treatment modalities such as LID.

## 5 Conclusion

Our study indicates that negative emotionality of an owner has a significant effect on his or her assessment of the hyperthyroid cat's QoL, but this effect is small, and therefore the Hyperthyroidism QoL‐cat tool can be used as a valid tool to assess the QoL of hyperthyroid cats. Based on the HRQoL assessment, RAIT is the treatment of choice, whereas LID or no treatment are the least favored options.

## Disclosure

Authors declare no off‐label use of antimicrobials.

## Ethics Statement

Authors declare no Institutional Animal Care and Use Committee or other approval was needed. Authors declare human ethics approval was not needed.

## Conflicts of Interest

The authors declare no conflicts of interest.

## Supporting information


**Data S1.** Supporting Information.
