# Supplementary material for: Influence of Owner Personality and Other Owner‐, Cat‐ and Treatment‐Related Factors on the Perception of Quality of Life in Cats With Hyperthyroidism
Source: J Vet Intern Med. 2025 Apr 15;39(3):e70091. doi: 10.1111/jvim.70091 (PMC11998023; doi:10.1111/jvim.70091)
Supplement: Supplementary file 1 — Data S1. Supporting Information. [file JVIM-39-e70091-s001.docx]

**Appendix 1:** **The HyperthyroidismCat-QoL** *(Blunschi, F., Schofield, I., Muthmann, S., Bauer, N. B., & Hazuchova, K. (2024). Development and validation of a questionnaire to assess health-related quality-of-life in cats with hyperthyroidism. Journal of Veterinary Internal Medicine, 1-24 https://doi.org/10.1111/jvim.1708)*

| HYPERTHYROIDISMCAT-QoL^7^ | | | |
| --- | --- | --- | --- |
| Owner related | | | |
| 1. Daily life | Due to your cat’s health condition and/ or its therapy, do you feel restricted in your daily life? | How often do you feel restricted by this? | - Very often - Never - Sometimes - Rarely - Never |
|  |  | How strongly are you affected by these restrictions? | - Very strongly - Strongly - Fairly - Insignificantly - Not at all |
| 2. Emotional burden | Does your cat’s health condition and/or its therapy impose an emotional burden on you? | How often do you feel emotionally burdened by this? | - Very often - Never - Sometimes - Rarely - Never |
|  |  | How strongly do you feel affected by this? | - Very strongly - Strongly - Fairly - Insignificantly - Not at all |
| 3. Financial burden | Does your cat’s health condition and/or its treatment impose a financial burden for you? | How often do you feel emotionally burdened by this? | - Very often - Never - Sometimes - Rarely - Never |
|  |  | How strongly do you feel emotionally burdened by this? | - Very strongly - Strongly - Fairly - Insignificantly - Not at all |
| 4. Medical treatment | Are you concerned about whether you are performing your cat’s medical treatments correctly? | How often are you concerned about this? | - Very often - Never - Sometimes - Rarely - Never |
|  |  | How strongly do you feel concerned about this | - Very strongly - Strongly - Fairly - Insignificantly - Not at all |
| 5. Side effects | Are you concerned about any side effects of the medical therapy that your cat could suffer from? | How often are you concerned about this? | - Very often - Never - Sometimes - Rarely - Never |
|  |  | How strongly do you feel concerned about this | - Very strongly - Strongly - Fairly - Insignificantly - Not at all |
| 6. Follow-ups | Are necessary medical follow-ups a burden for you? | How often do you feel burdened by this? | - Very often - Never - Sometimes - Rarely - Never |
|  |  | How strongly are you burdened by this? | - Very strongly - Strongly - Fairly - Insignificantly - Not at all |
| Cat related: Gastrointestinal, dietary, urination | | | |
| 7. Appetite | Have you noticed a change (increased or decreased) in your cat’s appetite? | How often does your cat show a changed appetite? | - Very often - Never - Sometimes - Rarely - Never |
|  |  | How pronounced is the change of your cat’s appetite? | - Very strongly - Strongly - Fairly - Insignificantly - Not at all |
| 8. Drinking behavior | Does your cat show a change in its drinking behavior? | How often does your cat show this change in behavior? | - Very often - Never - Sometimes - Rarely - Never |
|  |  | How pronounced is the change of your cat’s drinking behavior? | - Very strongly - Strongly - Fairly - Insignificantly - Not at all |
| 9. Weight | Does your cat show signs of persistent weight loss? | Does this statement apply to your cat? | - Yes - No |
|  |  | How pronounced is the weight change of your cat? | - Very strongly - Strongly - Fairly - Insignificantly - Not at all |
| 10. Nutritional condition | Does your cat show variations in its nutritional condition? | How often does this statement apply? | - Very often - Never - Sometimes - Rarely - Never |
|  |  | How pronounced are the changes in your cat’s nutritional condition? | - Very strongly - Strongly - Fairly - Insignificantly - Not at all |
| 11. Vomiting | Does your cat show vomiting? | How often does this statement apply? | - Very often - Never - Sometimes - Rarely - Never |
|  |  | How much do you feel this affects the quality of life of your cat? | - Very strongly - Strongly - Fairly - Insignificantly - Not at all |
| 12. Diarrhea | Does your cat suffer from diarrhea? | How often does this statement apply? | - Very often - Never - Sometimes - Rarely - Never |
|  |  | How much do you feel this affects the quality of life of your cat? | - Very strongly - Strongly - Fairly - Insignificantly - Not at all |
| 13. Urination | Does your cat show a change in urination behavior? | How often does this statement apply? | - Very often - Never - Sometimes - Rarely - Never |
|  |  | How much has your cat’s urination behavior changed? | - Very strongly - Strongly - Fairly - Insignificantly - Not at all |
| Cat related: Appearance | | | |
| 14. Fur quality | Is your cat’s fur less shiny? | Does this statement apply? | - Yes - No |
|  |  | How pronounced is this change in your cat’s fur? | - Very strongly - Strongly - Fairly - Insignificantly - Not at all |
| 15.Grooming behavior | Does your cat show decreased grooming behavior? | How often does this statement apply? | - Very often - Never - Sometimes - Rarely - Never |
|  |  | How strongly diminished is the grooming of your cat? | - Very strongly - Strongly - Fairly - Insignificantly - Not at all |
| 16. Muscle score | Does your cat show reduced strength/ muscling? | Does this statement apply? | - Yes - No |
|  |  | How strongly reduced is your cat in its strength/ muscling? | - Very strongly - Strongly - Fairly - Insignificantly - Not at all |
| Cat related: Behavior and emotions | | | |
| 17. Mood | Do you feel that your cat’s mood has altered through its health condition and/or its treatment? | How often does this statement apply? | - Very often - Never - Sometimes - Rarely - Never |
|  |  | How pronounced is this change in your cat’s mood? | - Very strongly - Strongly - Fairly - Insignificantly - Not at all |
| 18. Affection | Has your cat’s affection or attachment towards you changed? | How often does this statement apply? | - Very often - Never - Sometimes - Rarely - Never |
|  |  | How pronounced is this change in your cat’s affection or attachment? | - Very strongly - Strongly - Fairly - Insignificantly - Not at all |
| 19. Stress | Does your cat seem stressed more frequently? | How often does this statement apply? | - Very often - Never - Sometimes - Rarely - Never |
|  |  | How pronounced is this change in your cat’s behavior? | - Very strongly - Strongly - Fairly - Insignificantly - Not at all |
| 20. Sleeping behavior | Does your cat show an altered resting and sleeping behavior? | How often does this statement apply? | - Very often - Never - Sometimes - Rarely - Never |
|  |  | How pronounced is this change in your cat’s behavior? | - Very strongly - Strongly - Fairly - Insignificantly - Not at all |
| 21. Behavior | Is your cat increasingly withdrawn? | How often does this statement apply? | - Very often - Never - Sometimes - Rarely - Never |
|  |  | How pronounced is this change in your cat’s behavior? | - Very strongly - Strongly - Fairly - Insignificantly - Not at all |
| 22. Treatment stress | Does your cat seem stressed due to a required medical therapy? | How often does this statement apply? | - Very often - Never - Sometimes - Rarely - Never |
|  |  | How pronounced is this level of stress? | - Very strongly - Strongly - Fairly - Insignificantly - Not at all |
| 23. Playing behavior | Does your cat show a decreased playing behavior? | How often does this statement apply? | - Very often - Never - Sometimes - Rarely - Never |
|  |  | How strongly is the play behavior of your cat decreased? | - Very strongly - Strongly - Fairly - Insignificantly - Not at all |
| 24. Environment | Does your cat show less interest in its environment? | How often does this statement apply? | - Very often - Never - Sometimes - Rarely - Never |
|  |  | How strongly is the interest of your cat decreased? | - Very strongly - Strongly - Fairly - Insignificantly - Not at all |
| 25. Activity | Has your cat changed in its activity? | How often do you observe this change in behavior? | - Very often - Never - Sometimes - Rarely - Never |
|  |  | How pronounced is the change in your cat’s activity? | - Very strongly - Strongly - Fairly - Insignificantly - Not at all |
| General QoL | How would you rate your cat’s quality of life in general? |  | - Very poor - Poor - Satisfying - Good - Very good |

**Appendix 2: THE BIG FIVE INVENTORY-2 (BFI-2)**^34^ (Soto, C. J., & John, O. P. (2017). The next Big Five Inventory (BFI-2): Developing and assessing a hierarchical model with 15 facets to enhance bandwidth, fidelity, and predictive power. *Journal of Personality and Social Psychology*, *113*(1), 117–143. https://doi.org/10.1037/pspp0000096)

Here are a number of characteristics that may or may not apply to you. For example, do you agree that you are someone who *likes to spend time with others?* Please write a number next to each statement to indicate the extent to which you agree or disagree with that statement.

| **1** | **2** | **3** | **4** | **5** |
| --- | --- | --- | --- | --- |
| Disagree strongly | Disagree a little | Neutral; no opinion | Agree a little | Agree strongly |

1. I am someone who is outgoing, sociable.
2. I am someone who is compassionate, has a soft heart.
3. I am someone who tends to be disorganized.
4. I am someone who is relaxed, handles stress well.
5. I am someone who has few artistic interests.
6. I am someone who has an assertive personality.
7. I am someone who is respectful, treats others with respect.
8. I am someone who tends to be lazy.
9. I am someone who stays optimistic after experiencing a setback.
10. I am someone who is curious about many different things.
11. I am someone who rarely feels excited or eager.
12. I am someone who tends to find fault with others.
13. I am someone who is dependable, steady.
14. I am someone who is moody, has up and down mood swings.
15. I am someone who is inventive, finds clever ways to do things.
16. I am someone who tends to be quiet.
17. I am someone who feels little sympathy for others.
18. I am someone who is systematic, likes to keep things in order.
19. I am someone who can be tense.
20. I am someone who is fascinated by art, music, or literature.
21. I am someone who is dominant, acts as a leader.
22. I am someone who starts arguments with others.
23. I am someone who has difficulty getting started on tasks.
24. I am someone who feels secure, comfortable with self.
25. I am someone who avoids intellectual, philosophical discussions.
26. I am someone who is less active than other people.
27. I am someone who has a forgiving nature.
28. I am someone who can be somewhat careless.
29. I am someone who is emotionally stable, not easily upset.
30. I am someone who has little creativity.
31. I am someone who is sometimes shy, introverted.
32. I am someone who is helpful and unselfish with others.
33. I am someone who keeps things neat and tidy.
34. I am someone who worries a lot.
35. I am someone who values art and beauty.
36. I am someone who finds it hard to influence people.
37. I am someone who is sometimes rude to others.
38. I am someone who is efficient, gets things done.
39. I am someone who often feels sad.
40. I am someone who is complex, a deep thinker.
41. I am someone who is full of energy.
42. I am someone who is suspicious of others’ intentions.
43. I am someone who is reliable, can always be counted on.
44. I am someone who keeps their emotions under control.
45. I am someone who has difficulty imagining things.
46. I am someone who is talkative.
47. I am someone who can be cold and uncaring.
48. I am someone who leaves a mess, doesn’t clean up.
49. I am someone who rarely feels anxious or afraid.
50. I am someone who thinks poetry and plays are boring.
51. I am someone who prefers to have others take charge.
52. I am someone who is polite, courteous to others.
53. I am someone who is persistent, works until the task is finished.
54. I am someone who tends to feel depressed, blue.
55. I am someone who has little interest in abstract ideas.
56. I am someone who shows a lot of enthusiasm.
57. I am someone who assumes the best about people.
58. I am someone who sometimes behaves irresponsibly.
59. I am someone who is temperamental, gets emotional easily.
60. I am someone who is original, comes up with new ideas.

**Appendix 3**: **Table of questions concerning the socio-demographic data about the owner and information about the cat and its hyperthyroidism treatment**

| Question | Answer sets |
| --- | --- |
| CAT RELATED QUESTIONS | |
| 1. What is the name of your cat? | _______________________________________________ |
| 2. How old is your cat? | - Less than 6 months - 7 months to 2 years - 3 to 6 years - 7 to 10 years - 11 to 14 years - Over 15 years - Unknown |
| 3. What is the sex of your cat? | - Female - Female neutered - Male - Male neutered |
| 4. What breed is your cat? | - Domestic Shorthair/ European Shorthair - Maine Coon - Norwegian Forest Cat - British Shorthair - Bengal - Siamese - Persian Cat - Ragdoll - Mixed breed - Unknown - Other: _______________________ |
| 5. How long have you had your cat? | - Less than 6 months - 7 to 12 months - 1 to 2 years - 2 to 4 years - 5 to 10 years - For more than 10 years |
| 6. Is your cat an outdoor cat? | - No - Only on the leash. - Only in the garden and under supervision - Yes, my cat is an outdoor cat, but does come into the house - Yes, my cat is an outdoor cat and never comes into the house. |
| 7. For how long has your cat been hyperthyroid?  *Please state the time in months or years.* | _____________________________________________ |
| 8. How pronounced/ severe are the following symptoms in your cat?  *Please rate the symptoms on a scale from 1= not present to 10= very strong.*   \|  \| 1 \| 2 \| 3 \| 4 \| 5 \| 6 \| 7 \| 8 \| 9 \| 10 \| \| --- \| --- \| --- \| --- \| --- \| --- \| --- \| --- \| --- \| --- \| --- \| \| Weight loss \| o \| o \| o \| o \| o \| o \| o \| o \| o \| o \| \| Muscle wastage \| o \| o \| o \| o \| o \| o \| o \| o \| o \| o \| \| Restlessness \| o \| o \| o \| o \| o \| o \| o \| o \| o \| o \| \| Aggression \| o \| o \| o \| o \| o \| o \| o \| o \| o \| o \| \| Vomiting \| o \| o \| o \| o \| o \| o \| o \| o \| o \| o \| \| Diarrhea \| o \| o \| o \| o \| o \| o \| o \| o \| o \| o \| \| Increased urination \| o \| o \| o \| o \| o \| o \| o \| o \| o \| o \| \| Increased thirst \| o \| o \| o \| o \| o \| o \| o \| o \| o \| o \| \| Poor coat quality \| o \| o \| o \| o \| o \| o \| o \| o \| o \| o \| | |
| 9. How concerned are you about your cats Hyperthyroidism?  *Please rate the following issues on a scale from 1 = not a concern for me at all to 10= concerns me strongly.*   \| 1 \| 2 \| 3 \| 4 \| 5 \| 6 \| 7 \| 8 \| 9 \| 10 \| \| --- \| --- \| --- \| --- \| --- \| --- \| --- \| --- \| --- \| --- \| \| o \| o \| o \| o \| o \| o \| o \| o \| o \| o \| | |
| 10. What treatment options for hyperthyroidism are you aware of?  *Please select all options you have already heard of before you started this survey.* | - Antithyroid tablets or syrup (e.g. Felimazole, Thiamatab, Vidalta, Thyronorm, …) - Antithyroid ear ointment - Iodine reduced food (e.g.: Hills y/d) - Radioiodine therapy - Surgery/thyroidectomy - No therapy - Other: ______________________________ |
| 11. What therapy is your cat currently receiving for his/ her hyperthyroidism? | - Antithyroid tablets or syrup (e.g. Felimazole, Thiamatab, Vidalta, Thyronorm, …) - Antithyroid ear ointment - Iodine reduced food (e.g.: Hills y/d) - Radioiodine therapy - Surgery/ thyroidectomy - No therapy - Other: _______________________________ |
| 12. What was your main reason for choosing this treatment option? | - Advice from primary care veterinarian - Internet research - Personal advice (non-veterinarian) from a forum / discussion group - Advice from a friend - Own experience (my other cat has/had hyperthyroidism) - Own experience (I suffer from hyperthyroidism myself) - No therapy - Other: ____________________________________ |
| 13. For how long has your cat been receiving treatment for his/her hyperthyroidism? (In case of radioiodine therapy or thyroidectomy, please state how long ago this treatment was carried out.) | - Less than 1 months - 2 to 3 months - 4 to 6 months - 7 to 12 months - For about 1 year - For about 2 years - For about 3 years - For about 4 years - For about 5 years or longer - No therapy - Other: ____________________________________ |
| 14. Does your cat have normal thyroid levels at the moment? | - Yes - No, the thyroid hormone levels are increased. - No, the thyroid hormone levels are decreased. - I don't know. |
| 15. If your cat suffers from any of the above-mentioned diseases, what therapy is currently required? | - Oral medication (e.g., tablets) - Medical treatment with regular injections - Special diet - No current treatment - Other: _____________________ |
| 16. How often are the thyroid hormone levels of your cat being checked at the vet? | - Four times a year (every 3 months) - Three times a year (every 4 months) - Twice a year (every 6 months) - Once a year - Other: _____________________ |
| OWNER RELATED QUESTIONS | |
| 1. What year were you born? | _____________________ |
| 2. Please specify your gender. | - Male - Female - Divers - Prefer not to say - Other |
| 3. In which country are you currently resident? | _____________________ |
| 4. Are there any children living together with the cat? If yes, how old is the youngest child? | - No - Younger than 3 years - 3 to 6 years - 7 to 12 years - 13 to 18 years - Older than 18 years - Prefer not to say - Other: _____________________ |
| 5. What is your highest level of education? | - Secondary education - Bachelor’s degree - Master’s degree - State examination - Doctorate (PhD) - Prefer not to say - Other: |
| 6. What is your current work situation? | - Employed full-time - Employed part-time - Employed full-time (work from home) - Employed full-time (hybrid) - Unemployed (seeking work) - Unemployed (not seeking work) - Retired - Unable to work - Pupil - Student - Apprentice / trainee - Prefer not to say - Other: _____________________ |
| 7. Who cares mainly for your cat? | - You - Your partner - Other family members - Neighbors - Prefer not to say - Other: _____________________ |
| 8. How much time do you and your cat spend together in direct proximity (less than 2 meters apart)? | - I spend ____ hours per day with my cat. (Please estimate the time) - I don’t see my cat every day. - Other: _____________________ |
| 9. Is this cat your first cat? | - Yes - No |
| 10. Have you ever owned a cat with hyperthyroidism before? | - Yes - No |
| 11. How many cats do you own? | ____________________ |

**Appendix** **4: Socio-demographic data about the owners (n=500)**

| Question | Choice of options | Number n | % |
| --- | --- | --- | --- |
| Age | Up to 30 years | 39 | 7.8 |
|  | 31 to 40 years | 117 | 23.4 |
|  | 41 to 50 years | 113 | 22.6 |
|  | Over 50 years | 222 | 44.4 |
|  | Prefer not to say | 1 | 0.2 |
|  | No answer | 8 | 1.6 |
| Gender | Female | 470 | 94.0 |
|  | Male | 26 | 5.2 |
|  | No answer | 4 | 0.8 |
| Current country of residence | Germany | 131 | 26.2 |
|  | Austria | 6 | 1.2 |
|  | USA | 116 | 23.2 |
|  | United Kingdom | 44 | 8.8 |
|  | Australia | 11 | 2.2 |
|  | Canada | 14 | 2.8 |
|  | Other | 27 | 5.4 |
|  | No answer | 151 | 30.2 |
| Having children living in the same household (age of the youngest child) | No | 398 | 79.6 |
|  | Younger than 3 years | 11 | 2.2 |
|  | 3 to 6 years | 8 | 1.6 |
|  | 7 to 12 years | 22 | 4.4 |
|  | 13 to 18 years | 23 | 4.6 |
|  | Older than 18 years | 23 | 6.0 |
|  | Prefer not to say | 5 | 1.0 |
|  | Other | 3 | 0.6 |
| Highest level of education | Doctorate (PhD) | 13 | 2.6 |
|  | State examination | 26 | 5.2 |
|  | Master's degree | 67 | 13.4 |
|  | Bachelor's degree | 116 | 23.2 |
|  | Secondary education | 62 | 12.4 |
|  | Abitur or equivalent qualification | 45 | 9.0 |
|  | Realschule or equivalent qualification | 40 | 8.0 |
|  | Hauptschule or equivalent qualification | 6 | 1.2 |
|  | Prefer not to say | 13 | 2.6 |
|  | Other | 38 | 7.6 |
| Current work situation | Employed full-time | 178 | 35.6 |
|  | Employed part-time | 88 | 17.6 |
|  | Employed full-time (work from home) | 23 | 4.6 |
|  | Employed full-time (hybrid) | 52 | 10.4 |
|  | Unemployed (seeking work) | 10 | 2.0 |
|  | Unemployed (not seeking work) | 15 | 12.2 |
|  | Retired | 61 | 4.6 |
|  | Unable to work | 23 | 2.0 |
|  | Student | 10 | 0.2 |
|  | Apprentice/ Trainee | 1 | 0.2 |
|  | Prefer not to say | 9 | 1.8 |
|  | Other | 30 | 6.0 |
| Who cares mainly for the cat? | You | 448 | 89.6 |
|  | Your partner | 14 | 2.8 |
|  | Other family members | 5 | 1.0 |
|  | Neighbors | 0 | 0 |
|  | You and your partner | 25 | 5.0 |
|  | Prefer not to say | 0 | 0 |
|  | Other | 8 | 1.6 |
| Time spend together with the cat | More than 7 hours per day | 250 | 50.0 |
|  | Less than 7 hours per day | 226 | 54.2 |
|  | No answer | 21 | 4.2 |
|  | I don’t see my cat every day. | 3 | 0.6 |
| Is this cat the first cat | Yes | 102 | 20.4 |
|  | No | 398 | 79.6 |
| Owned a cat with hyperthyroidism before | Yes | 82 | 16.4 |
|  | No | 418 | 83.6 |
| How many cats are owned | One cat | 157 | 31.4 |
|  | Two cats | 157 | 31.4 |
|  | Three or more cats | 182 | 36.4 |
|  | No answer | 4 | 0.8 |

**Appendix** **5: The scores for the 15 subordinate facets of the BFI-2 and their comparison with the reference values of the German- and English-speaking population**

| **German-speaking population** | | | | | | | |
| --- | --- | --- | --- | --- | --- | --- | --- |
|  |  | **Female** | **Female reference value** | **Male** | **Male reference value** | **Total** | **Reference value** |
|  | N | 263 |  | 19 |  | 283 |  |
| **Extraversion** | **M** | **3.14** | **3.27** | **3.04** | **3.13** | **3.12** | **3.20** |
|  | **SD** | **0.65** | **0.63** | **0.55** | **0.61** | **0.65** | **0.62** |
| Sociability | M | 3.00 | 3.32 | 3.08 | 3.05 | 3.00 | 3.18 |
|  | SD | 0.92 | 0.83 | 0.81 | 0.78 | 0.91 | 0.82 |
| Assertiveness | M | 3.19 | 3.17 | 3.18 | 3.21 | 3.18 | 3.19 |
|  | SD | 0.86 | 0.76 | 0.68 | 0.73 | 0.84 | 0.75 |
| Energy level | M | 3.22 | 3.32 | 2.84 | 3.11 | 3.19 | 3.22 |
|  | SD | 0.76 | 0.69 | 0.76 | 0.68 | 0.77 | 0.69 |
| **Agreeableness** | M | **3.86** | **3.80** | **3.92** | **3.64** | **3.87** | **3.72** |
|  | SD | **0.52** | **0.49** | **0.44** | **0.51** | **0.51** | **0.51** |
| Compassion | M | 4.16 | 4.10 | **4.30** | **3.78** | **4.17** | **3.93** |
|  | SD | 0.64 | 0.62 | 0.54 | 0.66 | 0.63 | 0.66 |
| Respectfulness | M | 4.24 | 4.12 | **4.36** | **4.00** | 4.25 | 4.05 |
|  | SD | 0.59 | 0.58 | 0.53 | 0.60 | 0.58 | 0.59 |
| Trust | M | 3.19 | 3.17 | 3.09 | 3.15 | 3.18 | 3.15 |
|  | SD | 0.73 | 0.63 | 0.70 | 0.64 | 0.72 | 0.63 |
| **Conscientiousness** | M | **3.59** | **3.72** | **3.43** | **3.57** | **3.57** | **3.64** |
|  | SD | **0.56** | **0.62** | **0.70** | **0.59** | **0.58** | **0.61** |
| Organization | M | 3.62 | 3.75 | 3.47 | 3.60 | 3.61 | 3.67 |
|  | SD | 0.93 | 0.87 | 0.99 | 0.85 | 0.94 | 0.86 |
| Productiveness | M | 3.53 | 3.55 | 3.42 | 3.44 | 3.51 | 3.48 |
|  | SD | 0.75 | 0.74 | 0.81 | 0.70 | 0.76 | 0.71 |
| Responsibility | M | 3.61 | 3.87 | 3.39 | 3.66 | 3.60 | 3.75 |
|  | SD | 0.43 | 0.59 | 0.55 | 0.57 | 0.44 | 0.58 |
| **Negative  emotionality** | M | **2.89** | **2.82** | **2.84** | **2.66** | **2.88** | **2.74** |
|  | SD | **0.65** | **0.71** | **0.93** | **0.68** | **0.65** | **0.70** |
| Anxiety | M | 3.21 | 3.07 | **3.05** | **2.84** | **3.20** | **2.98** |
|  | SD | 0.84 | 0.71 | 0.81 | 0.71 | 0.84 | 0.72 |
| Depression | M | 2.60 | 2.63 | 2.60 | 2.56 | 2.61 | 2.61 |
|  | SD | 0.94 | 0.90 | 1.04 | 0.83 | 0.94 | 0.89 |
| Emotional volatility | M | 2.86 | 2.74 | 2.67 | 2.56 | 2.84 | 2.8 |
|  | SD | 0.54 | 0.82 | 0.55 | 0.79 | 0.54 | 0.80 |
| **Open-mindedness** | M | **3.54** | **3.39** | **3.73** | **3.28** | **3.55** | **3.34** |
|  | SD | **0.73** | **0.69** | **0.67** | **0.63** | **0.73** | **0.66** |
| Aesthetic sensitivity | M | 3.30 | 3.18 | 3.63 | 2.89 | 3.32 | 3.47 |
|  | SD | 1.01 | 0.98 | 1.27 | 0.96 | 1.03 | 0.71 |
| Intellectual curiosity | M | 3.62 | 3.46 | 3.82 | 3.46 | 3.63 | 3.02 |
|  | SD | 0.86 | 0.71 | 0.62 | 0.71 | 0.84 | 0.98 |
| Creative imagination | M | 3.71 | 3.51 | 3.74 | 3.50 | 3.71 | 3.50 |
|  | SD | 0.84 | 0.80 | 0.62 | 0.76 | 0.82 | 0.78 |
| **English-speaking population** | | | | | | | |
|  |  | **Female** | **Female reference value** | **Male** | **Male reference value** | **Total** | **Reference value** |
|  | N | 207 |  | 7 |  | 217 |  |
| **Extraversion** | **M** | **3.08** | **3.31** | **3.58** | **3.15** | **3.10** | **3.23** |
|  | **SD** | **0.74** | **0.80** | **0.92** | **0.78** | **0.75** | **0.80** |
| Sociability | M | 2.89 | 3.10 | 3.18 | 2.80 | 2.90 | 2.95 |
|  | SD | 1.04 | 1.07 | 1.38 | 1.02 | 1.05 | 1.05 |
| Assertiveness | M | 3.20 | 3.28 | 3.57 | 3.28 | 3.21 | 3.28 |
|  | SD | 0.92 | 0.93 | 0.80 | 0.92 | 0.92 | 0.93 |
| Energy level | M | 3.17 | 3.56 | 4.00 | 3.37 | 3.19 | 3.47 |
|  | SD | 0.82 | 0.89 | 1.19 | 0.88 | 0.84 | 0.89 |
| **Agreeableness** | M | **3.88** | **3.79** | **4.23** | **3.57** | **3.88** | **3.69** |
|  | SD | **0.56** | **0.60** | **0.29** | **0.65** | **0.56** | **0.64** |
| Compassion | M | 4.13 | 3.97 | 4.54 | 3.72 | 4.14 | 3.84 |
|  | SD | 0.71 | 0.76 | 0.44 | 0.79 | 0.71 | 0.78 |
| Respectfulness | M | 4.28 | 4.08 | 4.57 | 3.87 | 4.29 | 3.98 |
|  | SD | 0.65 | 0.68 | 0.51 | 0.73 | 0.64 | 0.71 |
| Trust | M | 3.22 | 3.32 | 3.57 | 3.13 | 3.22 | 3.23 |
|  | SD | 0.80 | 0.80 | 0.62 | 0.83 | 0.80 | 0.82 |
| **Conscientiousness** | M | **3.51** | **3.50** | **4.06** | **3.35** | **3.53** | **3.43** |
|  | SD | **0.63** | **0.79** | **0.76** | **0.74** | **0.64** | **0.77** |
| Organization | M | 3.51 | 3.51 | 4.00 | 3.33 | 3.52 | 3.42 |
|  | SD | 0.96 | 1.03 | 1.35 | 0.99 | 0.97 | 1.01 |
| Productiveness | M | 3.52 | 3.43 | 4.25 | 3.31 | 3.54 | 3.37 |
|  | SD | 0.83 | 0.93 | 0.92 | 0.87 | 0.84 | 0.90 |
| Responsibility | M | 3.51 | 3.57 | 3.93 | 3.40 | 3.53 | 3.48 |
|  | SD | 0.50 | 0.83 | 0.12 | 0.78 | 0.50 | 0.81 |
| **Negative  emotionality** | M | **2.95** | **3.18** | **2.37** | **2.95** | **2.94** | **3.07** |
|  | SD | **0.69** | **0.84** | **0.50** | **0.88** | **0.69** | **0.87** |
| Anxiety | M | 3.34 | 3.58 | 3.07 | 3.28 | 3.33 | 3.43 |
|  | SD | 0.91 | 0.88 | 0.84 | 0.95 | 0.91 | 0.93 |
| Depression | M | 2.69 | 2.88 | 1.89 | 2.82 | 2.67 | 2.85 |
|  | SD | 0.97 | 1.02 | 0.43 | 1.03 | 0.96 | 1.02 |
| Emotional volatility | M | 2.83 | 3.09 | 2.14 | 2.77 | 2.81 | 2.93 |
|  | SD | 0.60 | 1.04 | 0.43 | 1.04 | 0.60 | 1.05 |
| **Open-mindedness** | M | **3.67** | **3.91** | **4.06** | **3.93** | **3.68** | **3.92** |
|  | SD | **0.74** | **0.67** | **0.71** | **0.64** | **0.74** | **0.65** |
| Aesthetic sensitivity | M | 3.52 | 4.03 | 3.93 | 4.18 | 3.53 | 4.10 |
|  | SD | 0.99 | 0.71 | 0.47 | 0.69 | 0.99 | 0.70 |
| Intellectual curiosity | M | 3.72 | 3.88 | 4.04 | 3.71 | 3.73 | 3.80 |
|  | SD | 0.83 | 0.94 | 0.88 | 0.90 | 0.83 | 0.92 |
| Creative imagination | M | 3.78 | 3.82 | 4.21 | 3.89 | 3.79 | 3.85 |
|  | SD | 0.84 | 0.80 | 1.35 | 0.81 | 0.85 | 0.81 |

M=mean, SD=standard deviation
